# Sperm DNA Fragmentation Index and Hyaluronan Binding Ability in Men from Infertile Couples and Men with Testicular Germ Cell Tumor

**DOI:** 10.1155/2016/7893961

**Published:** 2016-11-24

**Authors:** Katarzyna Marchlewska, Eliza Filipiak, Renata Walczak-Jedrzejowska, Elzbieta Oszukowska, Slawomir Sobkiewicz, Malgorzata Wojt, Jacek Chmiel, Krzysztof Kula, Jolanta Slowikowska-Hilczer

**Affiliations:** ^1^Department of Andrology and Reproductive Endocrinology, Medical University of Lodz, Lodz, Poland; ^2^II Clinic of Urology, Medical University of Lodz, Lodz, Poland; ^3^Infertility Clinic, Salve Medica, Lodz, Poland

## Abstract

*Objective*. To investigate sperm DNA fragmentation and sperm functional maturity in men from infertile couples (IC) and men with testicular germ cell tumor (TGCT).* Materials and Methods*. Semen samples were collected from 312 IC men and 23 men with TGCT before unilateral orchiectomy and oncological treatment. The sperm chromatin dispersion test was performed to determine DNA fragmentation index (DFI) and the ability of sperm to bind with hyaluronan (HA) was assessed.* Results*. In comparison with the IC men, the men with TGCT had a higher percentage of sperm with fragmented DNA (median 28% versus 21%; *p* < 0.01) and a lower percentage of HA-bound sperm (24% versus 66%; *p* < 0.001). Normal results of both analyses were observed in 24% of IC men and 4% of men with TGCT. Negative Spearman's correlations were found between DFI and the percentage of HA-bound sperm in the whole group and in IC subjects and those with TGCT analyzed separately.* Conclusions*. Approximately 76% of IC men and 96% with TGCT awaiting orchiectomy demonstrated DNA fragmentation and/or sperm immaturity. We therefore recommend sperm banking after unilateral orchiectomy, but before irradiation and chemotherapy; the use of such a deposit appears to be a better strategy to obtain functionally efficient sperms.

## 1. Introduction

A number of factors, including chromosomal abnormalities, spermatogenic failure, urogenital infections, parental age, and those associated with a detrimental life style, have been linked with male infertility [1–3]. A clear relationship is known to exist between most of these situations and increased oxidative stress [4]. A positive relationship is known to exist between oxidative stress and DNA fragmentation in semen samples as indirect action [5]. Several studies have attempted to find an effective predictive marker for fertility. One of the most promising is assessment of DNA integrity. Several mechanisms responsible for DNA fragmentation are currently described [6]. According to the abortive apoptosis theory, the integrity of DNA depends on endonuclease activation in the presence of such apoptotic markers as the M450 bodies and cytoplasmic vacuoles in spermatozoa. Spermatogenic cells whose apoptotic process in the testis has not been completed might differentiate into sperm with fragmented DNA. Such a process generally leads to the production of double-stranded breaks in DNA. However, apoptosis occurring during spermatogenesis is not the only cause of abnormal DNA integrity. Another hypothesis that explains this phenomenon is the defective maturation theory, according to which, the physiological replacement of histones by protamines leads to sperm chromatin compaction, which might induce breakages in DNA. DNA integrity is also influenced strongly by topoisomerases; type II topoisomerases seem to be particularly responsible for both single- and double-stranded breaks during the removal of DNA supercoiling in elongated spermatids and failure of the religation process [7].

A serious problem with a known impact on fertility is testicular cancer [8]. In contrast to most solid cancers, which tend to occur in older people, testicular germ cell tumors (TGCT) develop predominantly in young men, with a peak prevalence at 18–34 years of age, this being the reproductive period of life [8, 9]. Although spermatogenic function is disturbed by oncological treatment, it may be restored in some cases [8, 10]. However, sometimes the only way to preserve fertility in these cases is by the cryopreservation of sperm samples before the treatment, which are later used in treatment with artificial reproductive technologies (ART). Although the use of ART, especially intracytoplasmic sperm injection (ICSI), has seen growing popularity, the efficiency of this treatment resulting in a child birth is only about 32%, even in couples with male partners not demonstrating any apparent sperm defect [11]. Two factors resulting in a restricted success rate of natural fertilization and ART are sperm DNA fragmentation and sperm-oocyte binding defects.

Diagnosis of male infertility is based mainly on the routine, basic analysis of sperm samples. It has become apparent that none of parameters assessed by the manual procedure or by Computer-Assisted Sperm Analysis (CASA) is sufficient for the determination of male fertility. Undoubtedly, there is an urgent need to develop new diagnostic methods to allow more specific evaluation of sperm quality.

One of the most promising methods is assessment of sperm DNA fragmentation. Fragmentation may occur in both single and double DNA strands and is observed particularly often in the ejaculate of subfertile men [12]. Recent studies on sperm DNA integrity indicate that semen samples containing a threshold value of sperm DNA fragmentation of more than 30% are associated with a decreased pregnancy rate and high loss of early pregnancy [4, 13].

Many assays are currently available for the measurement of sperm DNA fragmentation, that is, the sperm chromatin dispersion assay (SCDA) [14], the TUNEL (the terminal deoxynucleotidyl transferase-mediated dUDP nick end-labeling) assay [15], the comet assay (single-cell gel electrophoresis) [16], or the sperm chromatin structure assay (SCSA) [17]. A strong correlation has been found between the results of the TUNEL, SCDA, and SCSA tests [18].

Other sperm functional tests have been based on the assessment of the nuclear, membrane, and cytoplasmic maturity of spermatozoa. One test which is gaining popularity is the Hyaluronan Binding Assay (HBA), based on the observation that sperm binding to hyaluronic acid (HA) plays a critical role in the selection of mature, functionally competent spermatozoa during* in vivo *[19] and* in vitro* fertilization [20]. HA is a linear polysaccharide present in the extracellular matrix of cumulus oophorus around the oocyte. As HA is involved in the mechanism of sperm selection, only mature spermatozoa that have specific receptors for binding to HA can reach the oocyte interior and fertilize it [21, 22]. Spermatozoa that are able to bind with HA are mature and have completed the spermatogenic process of plasma membrane remodeling, cytoplasmic extrusion, and nuclear histone-protamine replacement. HA-bound sperm cells show intact acrosomes [21, 23]. A study of the characteristics of HA-bound spermatozoa indicated that they are devoid of cytoplasmic retention, persistent histones, DNA fragmentation, and apoptotic markers, such as caspase 3 [24], and are hence functionally mature. In contract, spermatozoa not bound to HA demonstrate low levels of HspA2, a testis-expressed chaperone which is part of the synaptonemal complex that directs and supports the meiotic process; they hence fail to undergo cytoplasmic membrane remodeling and are consequently unable to fertilize the oocyte. The expression of HspA2 has been shown to be lower in immature sperm with increased cytoplasmic retention [25].

The aim of the study was to investigate the degree of chromatin fragmentation in sperm and the ability of sperm to bind with HA in two different groups of men: one comprising men from infertile couples and the second comprising patients awaiting orchiectomy and oncological treatment following diagnosis for TGCT. This study stands in contrast to most similar studies, which investigate male gametes after orchiectomy but before cancer treatment.

## 2. Material and Methods

The study was approved by the Bioethical Committee of the Medical University in Lodz, Poland (no RNN/125/12/KE).

### 2.1. Participants

Semen samples were acquired from 312 men, aged 25–58 years (median 35), from infertile couples, and 23 men, aged 24–48 years (median 33), with TGCT before oncological treatment. The patients were referred to the laboratory of the Department of Andrology and Reproductive Endocrinology, Medical University of Lodz, in the years 2014-2015, to perform manual basic semen analysis according to WHO 2010 [26]. Two additional tests were performed: an assessment of sperm DNA fragmentation and an assessment of the number of sperm binding to HA.

Prior to the study, all patients received detailed information about the aim and method of the investigation and their consent was obtained.

Semen specimens were collected by masturbation after sexual abstinence of two to seven days.

### 2.2. Assessment of Sperm DNA Fragmentation

The SCDA is a simple, fast, and reliable assay developed for the determination of sperm DNA fragmentation [14]. In the present study, a Halosperm G2 Kit (Halotech, Madrid, Spain) was used.

Each sperm sample (50 *μ*L) was mixed gently with melted agarose (100 *μ*L) at 37°C. The sperm suspension was pipetted onto a microscopic slide, on a horizontal position, taking care to avoid air bubble formation, and covered with a coverslip. Samples were maintained at 4°C for five minutes before the coverslip was gently removed. Further processing was performed at room temperature. The samples underwent denaturation, lysis, washing, and dehydration processes according to the assay protocol. After drying, they were stained with the Diff-Quick reagent. Analysis was performed using NIS-Elements, Advanced Research version 3.21 (Nikon Instech, Tokyo, Japan) image analysis software. Samples were analyzed with a bright field microscope at a magnification of 600x (Nikon Eclipse E600, Tokyo, Japan).

**Figure 1:**
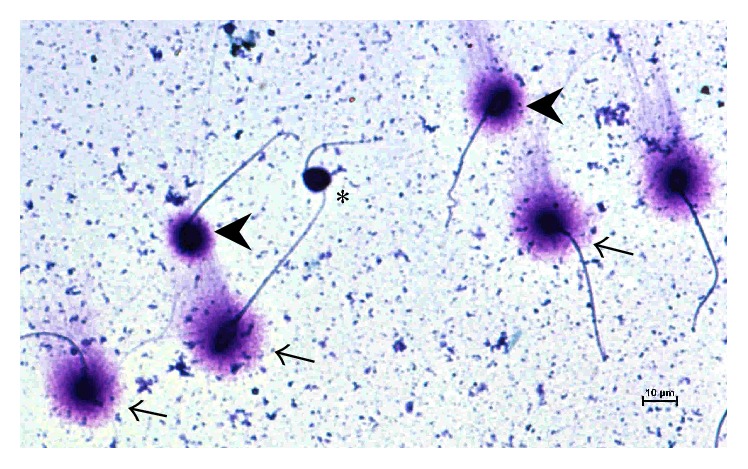
Representative microphotography of the sperm chromatin dispersion (SCDA) assay. The processed spermatozoa show large (arrow), medium (arrow head), and no (asterisk) halos of DNA dispersion under Diff-Quick staining. Bar 10 *μ*m.

Sperm cells were classified as follows (Figure 1):

1. sperm with nonfragmented DNA: spermatozoa with a large halo (halo width similar or greater than the minor diameter of the core) (arrows) and spermatozoa with a halo which is smaller but still greater than 1/3 of the minor diameter of the core (arrow heads);
2. sperm with fragmented DNA: spermatozoa with a halo size smaller than 1/3 of the minor diameter of sperm head and without a halo but with dark-stained core (asterisk); this group included sperm cells with degraded DNA and which presented a weakly stained core.

All analyses were performed in two replicates. Approximately 200 spermatozoa per replicate were assessed, with a total of 400 being counted.

The DNA Fragmentation Index (DFI) was calculated as the ratio of the number of spermatozoa with fragmented DNA to the number of all analyzed sperm cells. This value was given as a percentage. A DFI value of <30% was considered normal according to the manufacturer's suggestion [27].

### 2.3. Assessment of Sperm Binding with Hyaluronan Acid

The Hyaluronan Binding Assay (HBA) (Biocoat Inc., Horsham, PA, USA) was used to determine the proportion of sperm cells with the ability to bind with HA.

The HBA was carried out at room temperature. Each sample was stirred and 10 *μ*L was pipetted on the center of a special chamber coated with solid state hyaluronan, supplied with the kit. A CELL-VU-gridded coverslip was placed over the chamber, avoiding air bubble formation. The chamber was incubated at room temperature for 10–20 minutes, this time period being necessary for spermatozoa to bind to HA.

The samples were analyzed with a phase-contrast microscope (Nikon Eclipse E600, Tokyo, Japan) at a magnification of 400x. The numbers of bound motile spermatozoa and total motile spermatozoa were scored. Sperm motility was assessed by a manual method as described by the WHO (WHO, 2010). The ratio of HA-bound motile spermatozoa to all motile spermatozoa was calculated and shown as a percentage.

All analyses were performed in two replicates. Approximately 200 spermatozoa per replicate were assessed.

Proportions of HA-bound sperm cells greater than or equal to 80% were considered as normal, as recommended by the manufacturer of the assay. However, an HBA value <60% is considered by some other researchers as a better indicator of a high concentration of nonmature sperm cells with cytoplasmic retention [22].

### 2.4. Statistics

All analyses were performed using Statistica 12.0 for Windows (StatSoft Inc., Tulsa, OK, USA). The normality of the data distribution was analyzed using the Shapiro-Wilk test. As the results were distributed in a nonparametric manner, they were presented as median and range, and the ANOVA Kruskal-Wallis test was used to evaluate the difference between groups. Spearman's *r*-correlation coefficient was calculated between the results of the SCDA and HBA, as well as between the results of the SCDA, the HBA, and the age of patients. Differences were considered significant at *p* < 0.05.

## 3. Results

The results of the basic sperm analysis are presented in Table 1, as well as the age of the patients from the infertile couples and TGTC. The difference between the two groups of subjects was statistically significant for age and all sperm parameters. Results of sperm maturation, expressed by HA-binding ability, and sperm DNA fragmentation, expressed by DFI, are shown in Table 2.

Most men from infertile couples and men with TGCT revealed a decreased percentage of HA-bound sperm cells (<80%–72% and 96%, respectively; <60%–41% and 83%, resp.) and a relatively high level of DFI (≥30%–24% and 48%, resp.).

**Figure 2:**
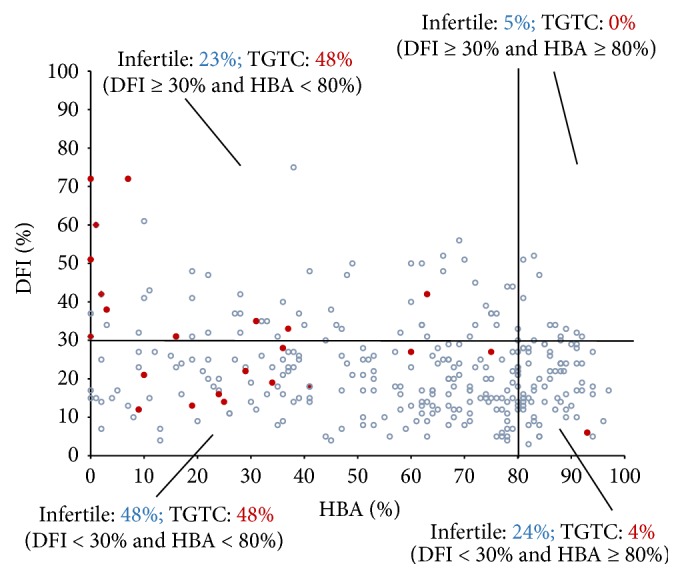
The proportion of men with a normal and increased DNA Fragmentation Index (DFI) and normal and decreased Hyaluronan Binding Assay (HBA) results of 312 patients from infertile couples (blue circles) and 23 patients with germ cell tumor (TGTC) (red circles). The vertical line corresponds to 80% of HA-bound sperm cells. The horizontal line corresponds to 30% of sperm with DNA fragmentation.

Of the group of men from infertile couples, 48% of samples demonstrated a low percentage of HA-bound sperm cells but normal sperm DFI results (Figure 2), 24% demonstrated normal values for both tests, and 23% had abnormal results for both tests. Finally, only 5% of the group demonstrated normal HBA results but an increased percentage of sperm cells with DNA fragmentation.

Only one patient with TGCT had normal results of both analyses (4%). The remaining patients had normal integrity of DNA and a decreased percentage of HA-bound sperm (48%) or abnormal results of both tests (48%) (Figure 2). There were no subjects with mature spermatozoa (HBA ≥ 80%) and fragmented DNA (DFI > 30%).

**Figure 3:**
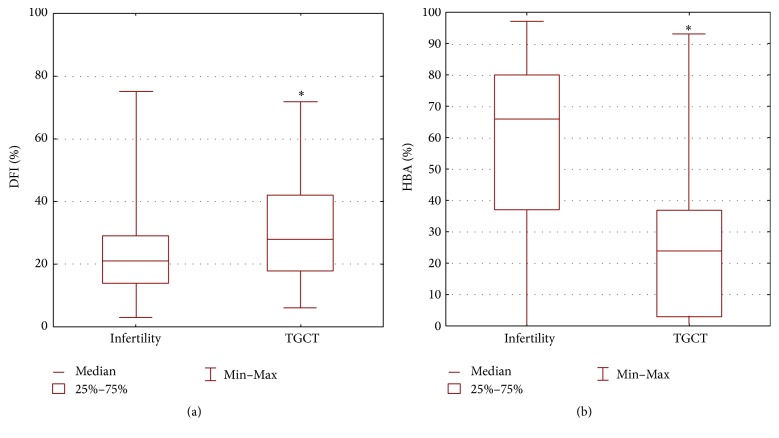
Comparison of (a) DNA Fragmentation Index (DFI) and (b) Hyaluronan Binding Assay (HBA) values between men from infertile couples and men with testicular germ cell tumor (TGCT). ANOVA Kruskal-Wallis: (a) *p* < 0.01 and (b) *p* < 0.001.

The median value of DFI was 21% in infertile patients (mean 22.7%; range 3–75%) and 28% in TGCT patients (mean 31.6%; range 6–72%) (Figure 3(a)). The median value of HBA was 66% in infertile patients (mean 57.8%; range 0–97%) and 24% (mean 26.7%; range 0–75%) in TGCT patients (Figure 3(b)).

Men with TGCT had significantly higher percentage of sperm cells with fragmented DNA and a significantly lower percentage of HA-bound sperm cells than the men from the infertile couples.

**Figure 4:**
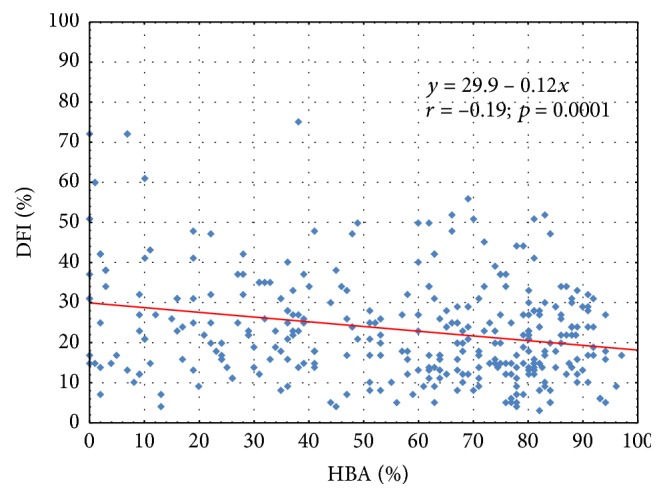
The correlation between the DNA Fragmentation Index (DFI) and results of Hyaluronan Binding Assay (HBA) in all analyzed patients.

Significant but weak negative correlations between HBA and DFI results were found throughout the whole investigated group (*r* = −0.19; *p* < 0.0001) (Figure 4), as well as in men from infertile couples (*r* = −0.15; *p* < 0.01), and moderate one in men with TGCT (*r* = −0.48; *p* < 0.05).

**Figure 5:**
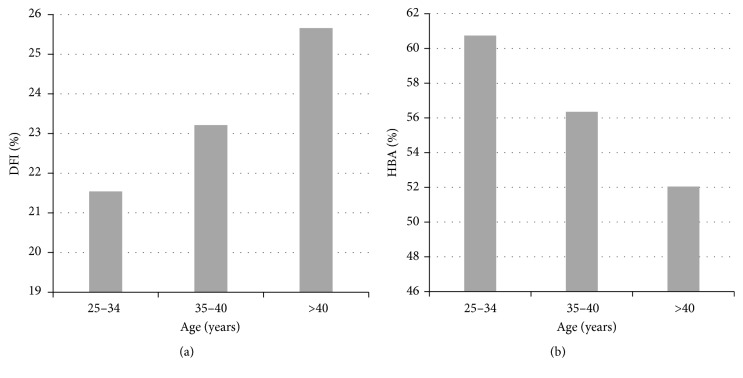
Comparison of DNA Fragmentation Index (DFI) (a) and the Hyaluronan Binding Assay (HBA) results (b) in 312 men of all ages from the infertile couples (without TGCT subjects).

The effect of age on sperm HA binding and sperm DNA fragmentation was also assessed in subjects from infertile couples. Although the percentage of HA-bound sperm was found to fall with age and the percentage of sperm with DNA fragmentation rises, no significant differences were observed between the different age groups (Figure 5).

## 4. Discussion

We present the results of an assessment of sperm DNA fragmentation and sperm binding with HA in two groups of men with a risk of defective sperm function: men from infertile couples and men diagnosed with TGCT awaiting orchiectomy and oncological treatment. Although the study included more men from infertile couples than those with TGCT, the incidence of TGCT is much less frequent [9].

Normal results for both analyses were observed in 24% of men from infertile couples but in only 4% of men with TGCT; however, it cannot be excluded that some men from the infertile couple might be fertile, that is, with a female factor of infertility. If so, this would result in a better DFI score and greater HA binding than would be found in a cohort of men from infertile couples with only male factor.

Until now few if any studies have examined the status of DNA fragmentation and HA binding in men diagnosed with testicular cancer awaiting orchiectomy treatment. The results reveal that 76% of the examined men from infertile couples experienced problems with sperm DNA fragmentation and/or sperm binding with HA but even 96% in TGCT group. Another study of chromatin integrity in 21 infertile and 15 postorchiectomy TGCT patients who were to undergo chemotherapy found mean DFI values (assessed by SCSA) to be significantly higher in both the infertile (22%) and TGCT patients (15%) in comparison to healthy controls (8%) [28]. Paoli et al. [29] report a mean DFI score of 18.0 ± 12.5% in a group of 131 postorchiectomy TGCT patients awaiting chemotherapy (assessed by SCSA). Similarly, Spermon et al. [30] report the median percentage of damaged sperm cells (assessed by TUNEL) to be 21% in postorchiectomy TGCT patients awaiting chemotherapy. Other studies show also that sperm DNA integrity may be damaged after orchiectomy [31, 32] and after oncological treatment [10, 33].

In the present study, the median value of sperm DFI, assessed by SCDA, was shown to be significantly higher in the group of TGCT patients (28%) than infertile men (21%). It should be emphasized that the analysis was conducted before orchiectomy; sperm cells produced in the testis with cancerogenesis were present in the ejaculate and their DNA may have been severely damaged. In addition, a high frequency of sperm chromatin fragmentation was found in patients with TGCT who were yet to receive any treatment, 48% patients with DFI ≥30%.

A significant negative correlation has been reported between sperm DNA damage and embryo quality* in vitro* and* in vivo* [34] and is connected with a higher risk of miscarriage [35]. Therefore, it is unclear whether to use semen cryopreserved before chemotherapy or fresh semen taken immediately after irradiation or chemotherapy, if present, for further fertility treatment [30]. Irradiation is a particularly dangerous factor inducing chromatin fragmentation [36], and exposure at a dose higher than 4 Gy may irreversibly affect spermatogenesis [37]. Paoli et al. (2015) report that chemotherapy and radiotherapy increased sperm DFI three to six months after the end of the treatment, improving after 12 to 24 months.

In the present study, the percentage of HA-bound sperm cells was found to be significantly lower in patients with TGCT than infertility patients, median/mean 24%/26.7% and 66%/57.8%, respectively. Molnar et al. (2014) note that, in samples taken from a group of 28 postorchiectomy TGCT patients, 56.9% of sperm cells were HA-bound (mean value). In addition, a decreased HA-binding capacity and a higher aneuploidy frequency were observed [38]. It can be assumed that sperm maturation is significantly impaired in the cancer patients, probably as a result of the process leading to the formation of neoplasia. In addition, a prospective, stratified, randomized, double-blinded, and controlled trial conducted in the USA found low HBA scores (≤65% of HA-bound sperm cells) in 318 of 802 (39.7%) tested couples from infertility clinics [20]. Comparable results were found in the present study, where 41% of samples taken from men from infertile couples contained <60% of HA-bound sperm cells. Therefore, there might be an elevated risk of transmitting chromosomal anomalies associated with the use of sperm from a patient with a low HA-binding score [39].

Our findings indicate that DNA fragmentation was only very rarely present in men with a high percentage of HA-bound sperm cells (≥80%). This has been confirmed by other authors who have demonstrated that the chromatin structure in the HA-bound sperm fraction tends to have high DNA integrity [40]. Our results indicated a weak, but significant, tendency for HA-bound sperm cells to have lower DFI scores, which confirms earlier findings based on a group of infertile patients [23]. This tendency suggests that poor sperm function may be attributed to a cause other than chromatin fragmentation. However, as DNA damage may arise during spermatogenesis from the failure to repair DNA breaks, the degree of damage would correlate well with other markers of spermatogenic failure, such as HA binding. Alternatively, if sperm DNA damage occurs as a result of the adverse effects of oxidative stress, then even mature spermatozoa with proper HA receptors may demonstrate DNA fragmentation.

Furthermore, in the present study, 48% of patients in both of the tested groups had semen with normal DFI but relatively small percentage of HA-bounded sperm cells (<80%), suggesting that poor sperm function may not necessarily be due to chromatin fragmentation. Nevertheless, as DNA fragmentation occurring during spermatogenesis may arise from a failure to repair DNA breakage, it would correlate well with other markers of spermatogenic failure, like spermatozoa binding with HA. However, if damage to sperm DNA is induced, albeit indirectly, by oxidative stress, even mature spermatozoa with proper HA receptors may show DNA fragmentation.

Although many animal and human studies have reported a correlation between DFI and age [41–43], no such significant relationship was revealed in the present study; however, a similar trend was observed. One probable cause is the nonparametric distribution of the age group. Our study group only included 15 young men under the age of 29 and 21 men over the age of 41 years. We can therefore assume that the effect of age on the presented analysis was minimal.

## 5. Conclusions

Our results indicate that 76% of men from infertile couples may have sperm DNA fragmentation and/or disturbed ability of sperm to bind with HA. The situation is worse in men with TGCT awaiting orchiectomy and oncological treatment, where 96% of cases may have poor DFI and HBA scores. Demonstration of the occurrence of sperm DNA fragmentation and poor sperm HA binding before orchiectomy and oncological treatment is a novel aspect of this study.

The results indicate that DFI and HBA assessment should be performed in men from infertile couples, especially when the cause of infertility is not diagnosed by routine diagnostics (idiopathic infertility). These assessments are also of importance in men with TGCT, both before and after cancer treatment, because the results of analyses could predict the potential of the sperm cells for fertilization. Based on our present findings and those of previous studies, we recommend sperm banking after orchiectomy but before irradiation and chemotherapy, as a better strategy to obtain functionally efficient sperm for ART.

## Figures and Tables

**Table 1:** Results of the age and conventional sperm parameters in 312 men from infertile couples and 23 men with testicular germ cell tumor (TGCT) presented as the median and range values.

Parameter	Infertile couples median (range)	TGTC median (range)
Age (year)	35 (25–58)	33 (24–48)
Volume (mL)	3.5 (0.7–9.8)	2.8 (0.9–6)
Sperm concentration (mln/mL)	33 (1–280)	6 (0.3–117)
Total sperm count (mln/ejaculate)	107 (2.9–868)	18 (1.3–326)
Progressive motility (%)	40.5 (3–76)	31 (3–61)
Total motility (%)	54 (5–85)	51 (5−82)
Normal morphology (%)	6 (0–19)	2 (1–10)

**Table 2:** Results of the assessment of sperm DNA fragmentation (DFI, DNA Fragmentation Index) and Hyaluronan Binding Assay (HBA) in 312 men from infertile couples and 23 men with testicular germ cell tumor (TGCT). *n*: number of patients.

Parameter	Men from infertile couples	Men with TGCT
	% (*n*)	% (*n*)
DFI < 30%	76.0 (237)	52.2 (12)
DFI ≥ 30%	24.0 (75)	47.8 (11)
HBA ≥ 80%	28.2 (88)	4.3 (1)
HBA < 80%	71.8 (224)	95.7 (22)
